# Society Meetings

**Published:** 1918-10

**Authors:** 


### SOCIETY MEETINGS

Brief reports and announcements of meetings of societies of Western New York, and those of general scope, are requested from Secretaries. Copy should be on hand the fifteenth of the month. Full transactions will be published at cost of composition.

**The Livingston Co. Medical Society** met at the Colonial Hospital at Dansville, Aug. 16. Dr. Shaw of Craig Colony was elected president, Dr. Wicker of Livonia vice-president, Dr. Collier of Craig Colony Sec. and Treas. The society has eight men in military service. The guests were entertained by the Dansville members.

The first meeting of the season of the **Western New York Medical Society** was held in Buffalo.. Dr. Lyman C. Lewis, secretary, resigned his office to join the army. Dr. Wm. E. Britt of Tonawanda was appointed to succeed him. The speakers were Major A. E. Brownrigg, commandant at For. Porter and Dr. Floyd M. Crandall of New York.

The thirteenth annual meeting of the **Eighth District Branch** of the New York State Medical Society was held in Buffalo. Dr. Albert T. Lytle, the president, addressed the meeting.

